# Improving the Catalytic Performance of Keggin [PW_12_O_40_]^3−^ for Oxidative Desulfurization: Ionic Liquids versus SBA-15 Composite

**DOI:** 10.3390/ma11071196

**Published:** 2018-07-12

**Authors:** Susana O. Ribeiro, Beatriz Duarte, Baltazar de Castro, Carlos M. Granadeiro, Salete S. Balula

**Affiliations:** 1 LAQV-REQUIMTE, Department of Chemistry and Biochemistry, Faculty of Sciences, University of Porto, 4169-007 Porto, Portugal; susananoribeiro@gmail.com (S.O.R.); beatriz-sm-duarte@hotmail.com (B.D.); bcastro@fc.up.pt (B.d.C.); 2 Department of Chemistry, CICECO—Aveiro Institute of Materials, University of Aveiro, Campus Universitário de Santiago, 3810-193 Aveiro, Portugal

**Keywords:** ionic liquids polyoxometalates, sustainable oxidative desulfurization, hydrogen peroxide, functionalized-SBA-15, benzothiophene derivatives

## Abstract

Different methodologies were used to increase the oxidative desulfurization efficiency of the Keggin phosphotungstate [PW_12_O_40_]^3−^ (PW_12_). One possibility was to replace the acid proton by three different ionic liquid cations, forming the novel hybrid polyoxometalates: [BMIM]_3_PW_12_ (BMIM as 1-butyl-3-methylimidazolium), [BPy]_3_PW_12_ (BPy as 1-butylpyridinium) and [HDPy]_3_PW_12_ (HDPy as hexadecylpyridinium. These hybrid Keggin compounds showed high oxidative desulfurization efficiency in the presence of [BMIM]PF_6_ solvent, achieving complete desulfurization of multicomponent model diesel (2000 ppm of S) after only 1 h, using a low excess of oxidant (H_2_O_2_/S = 8) at 70 °C. However, their stability and activity showed some weakness in continuous reused oxidative desulfurization cycles. An improvement of stability in continuous reused cycles was reached by the immobilization of the Keggin polyanion in a strategic positively-charged functionalized-SBA-15 support. The PW_12_@TM–SBA-15 composite (TM is the trimethylammonium functional group) presented similar oxidative desulfurization efficiency to the homogeneous IL–PW_12_ compounds, having the advantage of a high recycling capability in continuous cycles, increasing its activity from the first to the consecutive cycles. Therefore, the oxidative desulfurization system catalyzed by the Keggin-type composite has high performance under sustainable operational conditions, avoids waste production during recycling and allows catalyst recovery.

## 1. Introduction

The traditional technology for fuel desulfurization, hydrodesulfurization (HDS), has been widely used in refineries as it is highly efficient in the removal of aliphatic sulfur compounds from feedstock [1]. However, the process presents a high effective cost that is associated with the harsh experimental conditions that are necessary to achieve high efficiency (high temperature and pressure and high consumption of high quality hydrogen) [2]. Extractive and catalytic oxidative desulfurization (ECODS) is one of the most advantageous technologies for producing ultra-clean fuels due to its ability to efficiently remove the aromatic sulfur compounds from fuel under mild operating conditions [3]. The sulfur compounds are initially oxidized to polar compounds which can then be easily removed from the apolar oil phase by extraction with a polar solvent.

Over the last few years, numerous catalysts have been reported for ECODS application, such as mixed metal oxides [4,5], ionic liquids [6,7], metal–organic frameworks [8,9], titanium–zeolites [10,11,12], titanium-containing mesoporous silicas (SBA-15, MCM-41) [13,14] and polyoxometalates (POMs) [15,16]. The peculiar properties of POMs, such as their tunable acidity, solubility, thermal stability and resistance to oxidative decomposition has motivated their application in catalysis for decades [17].

Our research group has been developing several POM-based materials for applications in luminescence [18,19], gas separation [20], electrocatalysis [21,22], olefin oxidation [23,24] as well as oxidative desulfurization [25,26]. The preparation of organic POM hybrids has become a widespread methodology in POM chemistry [27], and in the case of catalysis, it has been used to enhance the efficiency and allow the separation of catalysts from reactional media [28,29]. Cationic surfactants, ionic liquids and copolymers have been used in the construction of organic POM hybrids, including those used for application in oxidative desulfurization [30,31,32,33,34]. Zhu et al. prepared a series of ionic liquid POM hybrids using different imidazolium cations and Keggin-type POMs [31]. The hybrids were tested as catalysts in the oxidation of dibenzothiophene (DBT) through an ECODS process. The authors showed that the catalytic activity is strongly influenced by the type of cations and metals, with the best catalysts achieving complete oxidation of DBT (S = 500 ppm) after 1 h.

In this work, two different kinds of catalysts based on the Keggin [PW_12_O_40_]^3−^ anion (PW_12_) were prepared. The first consisted of ionic liquid POM hybrids obtained by substitution of the starting protons of phosphotungstic acid by cations of ionic liquids (ILs). The ILs used were the bromide salts of 1-butyl-3-methylimidazolium (BMIM), 1-butylpyridinium (BPy) and hexadecylpyridinium (HDPy). The second kind of catalyst studied was a composite material obtained by the incorporation of PW_12_ into the mesoporous channels of positively-charged functionalized-SBA-15 (PW_12_@TM-SBA-15, TM is trimethylammonium). All the prepared catalysts were tested in the ECODS process of multicomponent model diesel containing the most refractory sulfur compounds in diesel. The desulfurization studies were performed using H_2_O_2_ as oxidant and an IL ([BMIM]PF_6_) or organic solvent (acetonitrile) as the extracting solvent. The influence of the solvents on the desulfurization performance was evaluated, and the reusability of the catalysts was investigated for consecutive ECODS cycles.

## 2. Experimental Section

### 2.1. Materials and Methods

The following chemicals and reagents were purchased from commercial suppliers and used without further purification: phosphotungstic acid hydrate (H_3_PW_12_O_40_·xH_2_O, Sigma-Aldrich, St. Loius, MO, USA), 1-butyl-3-methylimidazolium (BMIM) bromide (Fluka, Buchs, Switzerland, 97%), 1-butylpyridinium (BPy) bromide (Sigma-Aldrich, 99%), hexadecylpyridinium (HDPy) bromide (Sigma-Aldrich, 97%), Pluronic P123 (Aldrich), hydrochloric acid (HCl, Fluka), tetraethyl orthosilicate (TEOS, Sigma-Aldrich, 98%), *N*-trimethoxysilylpropyl-*N*,*N*,*N*-trimethylammonium chloride (TM, 50% in methanol, ABCR, Karlsruhe, Germany) and anhydrous toluene (Sigma-Aldrich, 99.8%). The reagents used for the ECODS studies were used as received, namely, dibenzothiophene (DBT, Sigma-Aldrich, 98%), 1-benzothiophene (1-BT, Fluka, 95%), 4-methyldibenzothiophene (4-MDBT, Sigma-Aldrich, 96%), 4,6-dimethyldibenzothiophene (4,6-DMDBT, Alfa-Aesar, Haverhill, MA, USA, 97%), *n*-octane (Sigma-Aldrich, 98%), tetradecane (Aldrich, 99%), acetonitrile (MeCN, Merck, Kenilworth, NJ, USA 99.5%), 1-butyl-3-methylimidazolium hexafluorophosphate ([BMIM]PF_6_, Sigma-Aldrich, 98%) and hydrogen peroxide (H_2_O_2_, Sigma-Aldrich, 30% *w*/*v* aq). Elemental analyses of C, N, and H were performed on a Leco CHNS-932 at the University of Santiago de Compostela. Infrared spectra were recorded in the 400–4000 cm^−1^ region on a Jasco 460 Plus Spectrometer (Jasco Analytical Instruments, Easton, PA, USA) using KBr pellets. ^31^P NMR spectra were collected for liquid solutions using a Bruker Avance III 400 spectrometer (Bruker, Freemont, CA, USA) and chemical shifts are given with respect to an external reference of 85% H_3_PO_4_. Scanning electron microscopy (SEM) and energy dispersive X-ray spectroscopy (EDS) studies were performed at the “Centro de Materiais da Universidade do Porto” (CEMUP, Porto, Portugal) using a JEOL JSM 6301F (JEOL Ltd., Tokyo, Japan) scanning electron microscope operating at 15 kV, equipped with an Oxford INCA Energy 350 energy-dispersive X-ray spectrometer (Oxford Instruments, Abingdon, UK). The samples were studied as powders and had previously been subjected to gold sputtering. Powder X-ray diffraction analyses were collected at ambient temperature in Bragg–Brentano para-focusing geometry using a Rigaku Smartlab diffractometer (Rigaku Co., Tokyo, Japan), equipped with a D/teX Ultra 250 detector and using Cu K-α radiation (K_α1_ wavelength 1.54059 Å) at 45 kV and 200 mA, in continuous mode, with a step of 0.01° and speed of 15°/min, in the range from 1 ≤ 2θ ≤ 50°. GC-FID analysis was carried out in a Bruker 430-GC-FID gas chromatograph to monitor the ODS experiments. Hydrogen was used as the carrier gas (55 cm^3^ s^−1^), and fused silica SPB-5 Supelco (Supelco Analytical, Bellefonte, PA, USA) capillary columns (30 m × 0.25 mm i.d.; 25 µm film thickness) were used.

### 2.2. Synthesis of Catalysts 

#### 2.2.1. Ionic Liquid–Polyoxometalates

The hybrids were prepared following an adaptation of the method by Zhang et al. [35]. An aqueous solution of H_3_[PW_12_O_40_]·*n*H_2_O (1 mmol in 5 mL) was added dropwise to a solution containing the ionic liquid (5 mmol). The BMIM and BPy ionic liquids were dissolved in water while HDPy was dissolved in acetonitrile. The mixture was stirred for 1 h at room temperature. The resulting solid was recovered by filtration, washed with water and dried in a desiccator over silica gel.

[BMIM]_3_PW_12_. Anal. Found (%): C, 8.89; N, 2.52; Calcd. (%) [C_8_H_15_N_2_]_3_(PW_12_O_40_)·nH_2_O (3295.55): C, 8.74, H, 1.38, N, 2.55. ^31^P (161.9 MHz, CD_3_CN, 25 °C): *δ* = −12.27 and −13.88 ppm. Selected FT-IR (cm^−1^): *ν* = 3465 (w), 3147 (m), 3114 (m), 2960 (m), 2931 (m), 2871 (m), 1562 (w), 1464 (w), 1385 (w), 1165 (m), 1080 (s), 978 (vs), 895 (s), 804 (vs), 746 (m), 650 (w), 621 (m), 596 (m), 521 (m); selected FT-Raman (cm^−1^): 3164 (w), 2958 (m), 2871 (w), 1562 (w), 1442 (m), 1414 (m), 1385 (w), 1336 (w), 1112 (w), 1023 (m), 1006 (vs), 991 (s), 918 (m), 826 (w), 517 (m), 472 (w). 

[BPy]_3_PW_12_. Anal. Found (%): C, 10.17; N, 1.22; Calcd. (%) [C_9_H_14_N]_3_(PW_12_O_40_)·nH_2_O (3286.52): C, 9.86, H, 1.29, N, 1.28. ^31^P (161.9 MHz, CD_3_CN, 25 °C): *δ* = −13.89 ppm. Selected FT-IR (cm^−1^): *ν* = 3435 (w), 3126 (w), 3086 (w), 3064 (w), 2966 (w), 2933 (w), 2875 (w), 1633 (m), 1487 (m), 1464 (w), 1317 (w), 1169 (w), 1080 (vs), 976 (vs), 897 (s), 802 (vs), 683 (s), 596 (w), 524 (m); selected FT-Raman (cm^−1^): 3093 (m), 2969 (m), 2937 (m), 2937 (m), 2875 (w), 1631 (m), 1581 (w), 1442 (m), 1309 (w), 1210 (w), 1167 (w), 1027 (s), 1005 (vs), 991 (s), 917 (m), 826 (w), 646 (m), 518 (m), 472 (w).

[HDPy]_3_PW_12_. Anal. Found (%): C, 20.49; N, 1.08; Calcd. (%) [C_21_H_38_N]_3_PW_12_O_40_·nH_2_O (3791.08): C, 19.94, H, 3.03, N, 1.11. ^31^P (161.9 MHz, CD_3_CN, 25 °C): *δ* = −14.13 ppm. Selected FT-IR (cm^−1^): *ν* = 3130 (m), 3087 (m), 3066 (m), 2922 (vs), 2850 (vs), 1633 (s), 1583 (w), 1500 (m), 1487 (s), 1466 (s), 1377 (w), 1354 (w), 1315 (w), 1215 (w), 1173 (m), 1080 (vs), 978 (vs), 895 (vs), 808 (vs), 766 (sh), 679 (vs), 594 (m), 521 (s), 509 (s); selected FT-Raman (cm^−1^): 3094 (m), 2891 (s), 2850 (s), 1633 (w), 1582 (w), 1438 (m), 1301 (w), 1214 (w), 1168 (w), 1028 (m), 1005 (vs), 991 (s), 918 (m), 646 (w), 517 (m), 472 (w).

#### 2.2.2. PW_12_@TM–SBA-15 Composite 

The SBA-15 support was initially functionalized with *N*-trimethoxyilylpropyl-*N*,*N*,*N*-trimethylammonium chloride (TM), as described in the literature [36]. The composite material was prepared through an impregnation method previously described by our group [37]. A solution of PW_12_ (1 g in 20 mL of water) was added to the functionalized support (TM–SBA-15, 0.5 g) and the mixture was stirred for 72 h. The solid was filtrated, washed thoroughly with water and dried in a desiccator over silica gel.

SBA-15. Anal. Found (%): N, 0.3; C, 4.5; H, 0.9. Selected FT-IR (cm^−1^): *ν* = 3400 (vw), 1652 (vw), 1198 (sh), 1070 (vs), 968 (m), 804 (m), 452 (vs); selected FT-Raman (cm^−1^): no significant Raman bands are observed.

TM–SBA-15. Anal. Found (%): N, 1.4; C, 7.6; H, 2.2; 0.098 mmol of TM per g of material. Selected FT-IR (cm^−1^): *ν* = 3736 (w), 2360 (m), 2342 (m), 1196 (sh), 1068 (vs), 952 (w), 804 (m), 668 (m), 446 (vs); selected FT-Raman (cm^−1^): 3028 (vs), 2972 (vs), 2934 (vs), 2893 (s), 2825 (w), 1451 (s), 911 (m), 753 (m).

PW_12_@TM–SBA-15. Anal. Found (%): N, 1.5; C, 7.7; H, 1.8; W, 12.3%; Si, 3.8%. loading of PW_12_ = 0.056 mmol g^−1^. Si/W (molar) = 2.0; ratio of TM/POM = 19.1. Selected FT-IR (cm^−1^): *ν* = 3435 (m), 2939 (sh), 1655 (m), 1508 (w), 1388 (w), 1192 (sh), 1082 (vs), 951 (s), (m), 901 (w), 808 (s), 741 (sh), 667 (w), 459 (s); selected FT-Raman (cm^−1^): 3031 (m), 2971 (s), 2936 (s), 2894 (m), 1448 (m), 1415 (m), 1348 (w), 1007 (vs), 990 (s), 912 (m), 864 (w), 749 (w), 516 (m).

### 2.3. Extractive and Catalytic Oxidative Desulfurization (ECODS) Process

The ECODS studies were performed using multicomponent model diesel containing 2000 ppm of sulfur content dissolved in *n*-octane. This solution was composed by approximately 500 ppm of dibenzothiophene (DBT), 500 ppm of 1-benzothiophene (1-BT), 500 ppm of 4-methyldibenzothiophene (4-MDBT) and 500 ppm of 4,6-dimethyldibenzothiophene (4,6-DMDBT). A biphasic system was composed of equal volumes of model diesel and extraction solvent (0.75 mL each one). The process began with an initial extraction in the presence of the catalyst (3 µmol) with stirring for 10 min at 70 °C. The catalytic stage was then initiated with the addition of aqueous H_2_O_2_ 30% (0.35 mmol). The quantification of each sulfur compound present in the model diesel phase was performed periodically (S_p_) by gas chromatography, periodically removing an aliquot of diesel from the system and adding an aliquot of tetradecane solution (in n-octane) as a standard. The percentage of desulfurization (Des) was determined for each sulfur compound relative to the initial sulfur concentration (S_initial_) in the model diesel; therefore, Des = (S_initial_ − S_p_)/S_initial_ × 100. The reusability of the catalysts was evaluated by removing the desulfurized model diesel at the end of an ECODS cycle and adding a new portion of untreated model diesel and oxidant.

## 3. Results and Discussion

### 3.1. Catalyst Characterization

**Figure 1:**
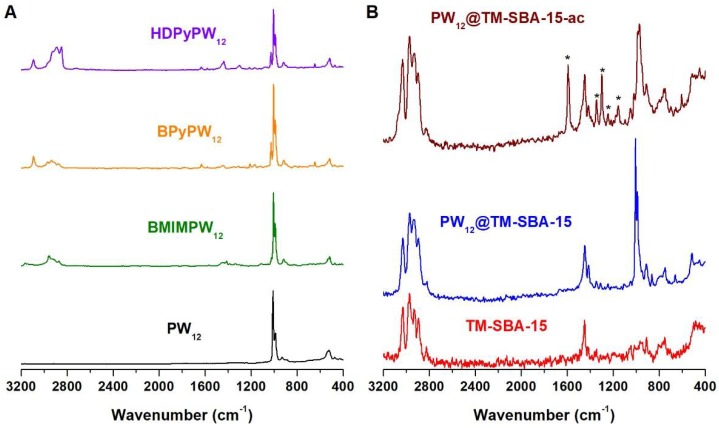
FT-Raman spectra of (**A**) the PW_12_-hybrids and (**B**) the amine-functionalized TM–SBA-15 and the corresponding PW_12_@TM–SBA-15 composite before and after catalysis. Asterisks (*) denote bands associated with organosulfur compounds that remained adsorbed to catalysts.

Different catalysts were prepared based on the Keggin [PW_12_O_40_]^3−^ anion (PW_12_), namely, ionic liquid–PW_12_ (IL–PW_12_) hybrids and a composite material. The hybrids were prepared by replacing the protons in phosphotungstic acid by the cations of the ionic liquids, 1-butyl-3-methylimidazolium (BMIM), 1-butylpyridinium (BPy) and hexadecylpyridinium (HDPy). The number of ionic liquid cations in the hybrids was determined by elemental analysis. The composite material was obtained through the incorporation of PW_12_ into the mesoporous channels of amine-functionalized-SBA-15. The vibrational spectra of IL–PW_12_ (Figure 1A and Figure S1A in Supplementary Materials) exhibit the characteristic bands associated with anionic PW_12_, together with the bands ascribed to its cationic counterpart. In all spectra, the bands associated with PW_12_ stretching modes can be clearly observed in the 1100–800 cm^−1^ range, namely, *ν*(P–O), terminal *ν*(W=O), corner-shared *ν*(W–O_b_–W) and edge-shared *ν*(W–O_c_–W) by decreasing wavenumber [38,39,40]. The bands associated with the ionic liquid cations can be observed in the 3164–3064 cm^−1^ and 2970–2850 cm^−1^ ranges, corresponding to *ν*(C–H) of the aromatic heterocycles and aliphatic chains, respectively [29,41]. Moreover, the bands located in the 1173–1165 cm^−1^ range, which are clearer in the FT-IR spectra, can be assigned to the *δ*(H–C–C) and *δ*(H–C–N) modes in the heterocycles [31,41,42]. Regarding the PW_12_@TM–SBA-15 composite, the FT-IR spectrum (Figure S1B in Supplementary Materials) is dominated by the intense bands associated with the SBA-15 support, namely the *ν*_as_(Si–O–Si), *ν*_s_(Si–O–Si) and *δ*(O–Si–O) vibrational modes, located at 1082, 808 and 459 cm^−1^, respectively [43,44]. Some of the PW_12_ vibrational modes are occluded by the intense silica bands. However, the appearance of an additional band at 951 cm^−1^ and the increased relative intensity of the band at 808 cm^−1^, which can be assigned to the *ν*(W=O) and *ν*(W–O_c_–W) stretches, respectively, point to the presence of PW_12_ in the composite material. The FT-Raman is an extremely useful technique for the characterization of siliceous-based composites due to their relatively weak Raman signals [19,45,46]. Therefore, the presence of PW_12_ on the composite is more evident in the FT-Raman spectrum, since the FT-Raman spectrum of PW_12_@TM–SBA-15 (Figure 1B) exhibits very intense bands in the 1010–860 cm^−1^ range that are associated with the characteristic PW_12_ vibrations [38,47]. The spectrum also displays the bands arising from the presence of amine groups, namely, the *ν*(C–H) and *δ*(CH_2_) vibrational modes in the 3031–2890 cm^−1^ and 1450–1410 cm^−1^ ranges, respectively [36,48]. The successful preparation of PW_12_@TM–SBA-15 was further confirmed by an elemental analysis which showed a PW_12_ loading of 0.056 mmol/g.

**Figure 2:**
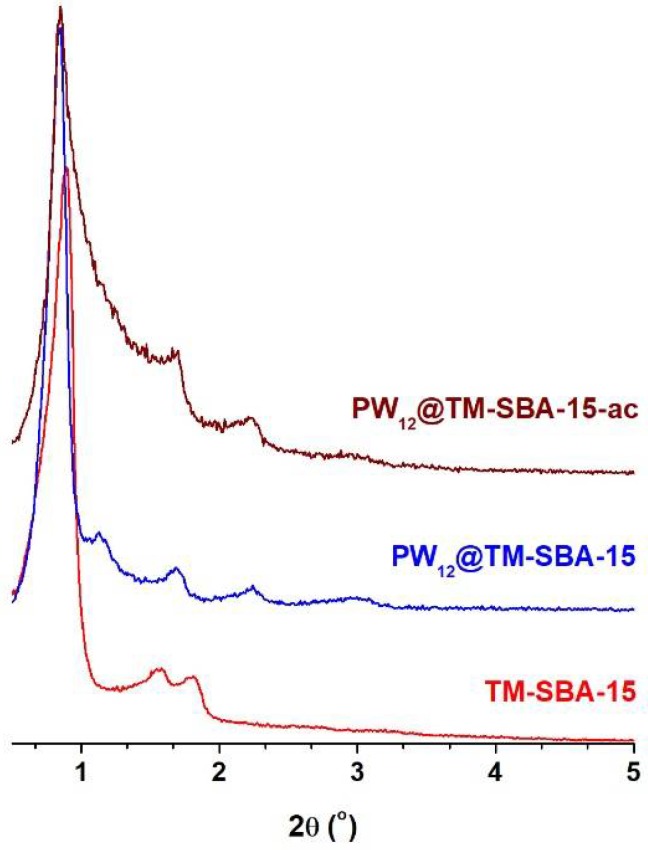
Powder XRD patterns of amine-functionalized SBA-15 (TM–SBA-15) and the corresponding PW_12_@TM–SBA-15 composite before and after catalysis (abbreviated as ac).

The PW_12_@TM–SBA-15 composite and support were analyzed by powder XRD (Figure 2). The TM–SBA-15 pattern exhibited the typical low-angle three peaks of SBA-15 materials which can be indexed as (100), (110) and (200) reflections of a *p*6*mm* hexagonal symmetry [49,50]. After the PW_12_ incorporation, a shift to higher 2θ can be observed in the PW_12_@TM–SBA-15 pattern, in particular, for the peaks assigned to the (110) and (200) reflections. Previous works dealing with POM-incorporated SBA-15 materials have reported this shift to higher angles which has been attributed to the occupancy of SBA-15 channels by the guest species [37,44,51,52]. 

**Figure 3:**
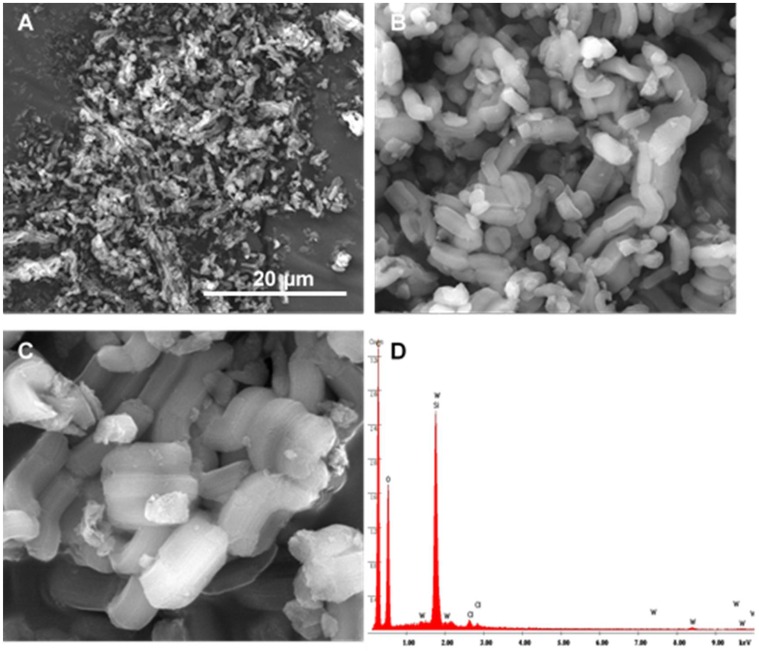
SEM images of the PW_12_@TM–SBA-15 composite material at different magnifications: (**A**) ×5000; (**B**) ×25,000; (**C**) ×60,000 and (**D**) energy dispersive X-ray spectroscopy (EDS) spectrum.

The SEM images of PW_12_@TM–SBA-15 (Figure 3) reveal that the morphology of the starting support is retained in the final composite. The images show hexagonal particles assembled in elongated structures which are typical of the mesoporous SBA-15 framework [44,49,53]. The chemical composition of PW_12_@TM–SBA-15 was evaluated by EDS spectroscopy (Figure 3D). The spectrum obtained is mainly composed of an intense peak assigned to silicon from the SBA-15 support but also by peaks assigned to tungsten which is consistent with the presence of the PW_12_ in the composite material.

### 3.2. Extractive and Catalytic Oxidative Desulfurization (ECODS) 

The oxidative desulfurization studies were performed using a model diesel containing the representative refractory sulfur compounds in diesel: approximately 500 ppm or 0.0156 mol dm^−3^ of 1-benzothiophene (1-BT), dibenzothiophene (DBT), 4,6-dimethyldibenzothiophene (4,6-DMDBT) and 4-methyldibenzothiophene (4-MDBT) in n-octane. The ECODS of model diesel was carried out in the presence of an extraction solvent with a ratio of 1:1 and in the presence of H_2_O_2_ as an oxidant. Two different extraction solvents were tested: acetonitrile (MeCN) and an ionic liquid (IL), 1-butyl-3-methylimidazolium hexafluorophosphate ([BMIM]PF_6_). The negligible vapour pressure of ILs makes them an appealing alternative to conventional volatile solvents, such as MeCN, especially when working below the decomposition temperature of ILs. However, the price and availability of ILs are still major drawbacks for their large scale application. Therefore, a compromise should be made between cost and toxicity when choosing an adequate solvent for ECODS. The ECODS system is formed by two main steps: the initial extraction and the catalytic stage. Initially, the extraction of the non-oxidized sulfur compounds from the model diesel to the extraction phase occurs during a 10 min period at 70 °C. After this time, the distribution of sulfur compounds between the two phases achieves equilibrium and the desulfurization of the model diesel stops. To continue the desulfurization of the model diesel, the oxidant H_2_O_2_ was added to the system (H_2_O_2_/S = 8) to oxidize the sulfur components present in the extraction phase into the corresponding sulfones and/or sulfoxides to promote a continuous transfer of more sulfur compounds from the model diesel to the extraction phase. No oxidized products were detected in the model diesel phase suggesting that the catalytic oxidative reaction should only occur in the extraction (MeCN and [BMIM]PF_6_) phase. The ECODS system was catalyzed by three different homogeneous catalysts based in ionic liquids of Keggin polyanion [PW_12_O_40_]^3−^ (abbreviated as PW_12_). These IL–PW_12_ compounds have distinct organic cations: 1-butyl-3-methylimidazolium ([BMIM]_3_PW_12_), 1-butylpyridinium ([BPy]_3_PW_12_) and hexadecylpyridinium ([HDPy]_3_PW_12_). A heterogeneous catalyst based on the same catalytic active center, PW_12_, immobilized on trimethylammonium-functionalized SBA-15 support (PW_12_@TM–SBA-15) was also used. 

#### 3.2.1. ECODS Using Homogeneous IL–PW_12_

**Figure 4:**
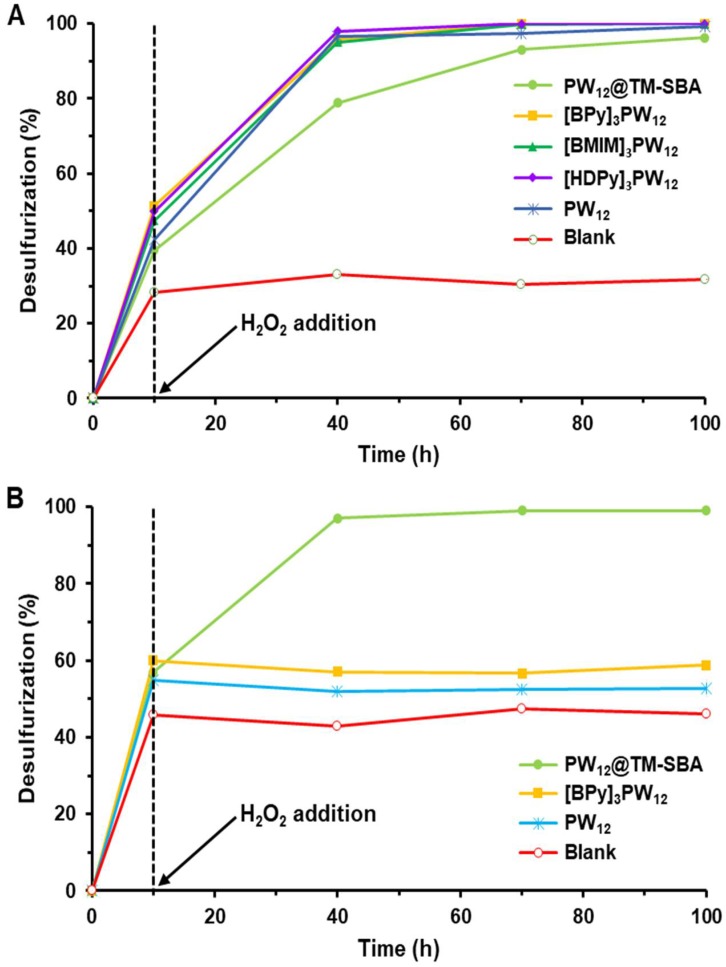
Kinetic desulfurization profiles of the extractive and catalytic oxidative desulfurization (ECODS) process catalyzed by PW_12_, IL–PW_12_ compounds, composite material and blank experiments (without catalyst) using (**A**) [BMIM]PF_6_ and (**B**) MeCN as extraction solvents at 70 °C.

Initially, a comparative study was performed between the different IL–PW_12_ compounds using both biphasic systems: model diesel/MeCN and model diesel/[BMIM]PF_6_. Figure 4 displays the desulfurization results obtained using MeCN and [BMIM]PF_6_ extraction solvents. It is possible to verify that the activity of the three IL–PW_12_ compounds is similar using the [BMIM]PF_6_ extraction solvent (Figure 4A). Figure 4B demonstrates that the [BPY]PW_12_ catalyst did not promote any oxidation using the model diesel/MeCN ECODS system, since the desulfurization stopped after the initial extraction step (after the first 10 min). On the other hand, using the model diesel/[BMIM]PF_6_ system, the three IL–PW_12_ catalysts achieved complete desulfurization after 1 h of oxidation (Figure 4A). Only slightly inferior activity was observed using PW_12_ as the precursor; this is probably due to the fast cationic exchange with the IL cation, according to the preparative method of hybrid IL–PW_12_, described in Section 2.2.1. By comparing the results obtained with [BMIM]PF_6_ and MeCN solvents, it is possible to confirm that the [BMIM]PF_6_ as extraction solvent has a collaborative performance when activating the catalyst, since, in the absence of IL–PW_12_, the ECODS systems did not promote any oxidative desulfurization (Figure 4A). This behavior has been observed previously in the literature where [BMIM]_3_PW_12_ was used as a catalyst for the epoxidation of olefins, and low activity was found in the presence of MeCN solvent and a high catalytic performance was observed using [BMIM]PF_6_ IL solvent [54]. In fact, this IL can be used not only as a solvent, but also should supply a special environment that facilitates the formation of the active peroxotungstate compounds through the interaction of IL–PW_12_ and H_2_O_2_. In fact, the literature suggests that the mechanism for the oxidation of benzothiophene derivatives catalyzed by polyoxometalates (POMs) and using H_2_O_2_ as an oxidant starts with the formation of active species through the interaction of the oxidant (H_2_O_2_) and the W^VI^ atoms of the POM (PW_12_ in this study) [16,26,46,55,56,57,58]. The resulting hydroperoxy- or peroxo-POM species are able to oxidize the sulfur compounds into the corresponding sulfoxides through a nucleophilic attack. The subsequent oxidation of the sulfoxides leads to the formation of sulfones. The oxidation promotes the continuous mass transport of sulfur compounds from the model diesel into the extraction phase ([BMIM]PF_6_) in order to restore the equilibrium of the extraction process. After 2 h of ECODS processes, the IL extraction phase was also analyzed by GC, and only sulfones and a vestigial amount of 1-BT sulfoxide was detected.

**Figure 5:**
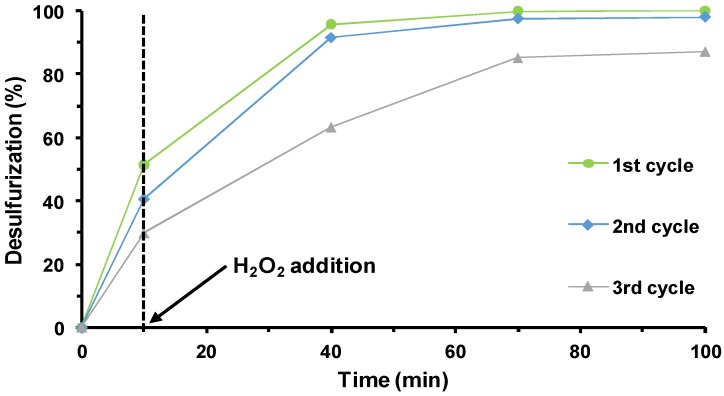
Kinetic desulfurization profiles catalyzed by [BPy]_3_PW_12_ for three consecutive ECODS cycles using ionic liquid ([BMIM]PF_6_) as extraction solvent at 70 °C.

In our previous works, it was possible to demonstrate the reusability of POM@[BMIM]PF_6_ systems in various consecutive cycles [40,59]. In these works, the ECODS systems were recycled by washing the IL phase with a mixture of strategic organic solvents to remove the oxidized and non-oxidized sulfur compounds. More recently, our group published a successful reused system that performs by only replacing the desulfurized diesel with new sulfurized diesel and a new aliquot of oxidant [25]. The same procedure was adopted in this work using the [BPY]_3_PW_12_@[BMIM]PF_6_ system, i.e., the [BPY]_3_PW_12_ compound immobilized in the IL extraction phase. This can be considered a continuous recycling system without the need for organic polar solvent usage and without the possibility of leaching active species during the clean IL process. Since the catalytic activities of the different IL–PW_12_ compounds were similar, the reusability was only performed for one of the three hybrid compounds ([BPY]_3_PW_12_). Figure 5 presents the reused data for three consecutive ECODS cycles. It is possible to observe that the catalytic performance of the [BPY]_3_PW_12_@[BMIM]PF_6_ system was essentially maintained from the first to the second cycle. However, from the second to the third cycle, a decrease in desulfurization was observed. In fact, a decrease in the initial extraction, i.e., the desulfurization obtained during the first 10 min of stirring at 70 °C, decreased from the first to the second cycle and also from the second to the third cycle. This is probably due to the number of oxidized sulfur compounds accumulated in the extraction phase ([BMIM]PF_6_ phase) over the reusability cycles that decelerate the transfer of more non-oxidized sulfur compounds. This phenomenon also promotes the lower oxidative catalytic activity of the IL–PW_12_ catalyst. On the other hand, a leaching of the active homogeneous catalyst [BPY]_3_PW_12_ to the model diesel phase can also promote a decrease in oxidative desulfurization efficiency. Therefore, the model diesel phase was analyzed by ^31^P NMR and by UV-Vis spectroscopy (a characteristic transition charge band at approximately 250 nm can be observed in the presence of Keggin-type polyoxometalate), but the presence of [BPY]_3_PW_12_ in the model diesel phase was not detected. These results suggest the absence of catalyst leaching from the [BMIM]PF_6_ phase. 

#### 3.2.2. ECODS Using Heterogeneous PW_12_@TM–SBA-15

A new heterogeneous catalyst was prepared by the strategic immobilization of Keggin structure PW_12_ into positively-charged functionalized-SBA-15 (TM–SBA-15). The SBA-15 has proven to be a suitable support to immobilize POMs when functionalized with appropriate functional groups [44,51,60,61]. In addition, this support has demonstrated that it can be used efficiently in oxidative desulfurization processes [51,54,62,63,64,65,66,67]. In this work, the trimethylammonium functional group was strategically selected to immobilize effectively the anionic PW_12_ by ionic interaction. The preparation of PW_12_@TM–SBA-15 allows the straightforward removal of catalysts from the ECODS system. 

**Figure 6:**
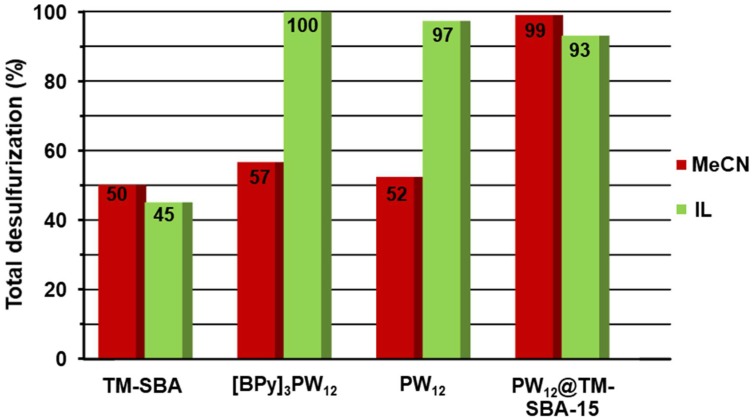
Desulfurization data of multicomponent model diesel obtained after 1 h in the presence of the support (TM–SBA), [BPy]_3_PW_12_, PW_12_ and PW_12_@TM–SBA-15 with MeCN or IL ([BMIM]PF_6_) as extraction solvent.

The ECODS studies were performed using this heterogeneous catalyst under the same conditions that were previously presented for the homogeneous IL–PW_12_. The model diesel was desulfurized using MeCN and [BMIM]PF_6_ extraction solvents (Figure 4). Contrary to what was observed with IL–PW_12_ catalysts, the catalytic activity of the composite PW_12_@TM–SBA-15 was considered similar in the presence of MeCN and IL extraction solvents. A difference in desulfurization efficiency observed during the first 30 min of the oxidation step was attributed to the lower initial extraction rate from using the IL extraction solvent (39% using IL instead of 57% using MeCN, Figure 4 and Table S1 in Supplementary Materials). Based on the superior activity of the composite compared to the IL-PW_12_ using the model diesel/MeCN system, the catalytic contribution of the support TM–SBA-15 was investigated (Figure 6 and Figure S2 in Supplementary Materials); however, it was demonstrated that this support material does not have any oxidative catalytic performance, since the desulfurization stopped after the initial extraction process, even in the presence of excess H_2_O_2_ oxidant, using either MeCN or IL extraction solvents. Using the model diesel/IL ECODS system it was possible to observe that desulfurization profile of the composite is slightly lower than that of the IL–PW_12_ homogeneous catalysts, since complete desulfurization was achieved after 1.5 h using the composite and 1 h using the IL–PW_12_ catalysts. By comparing the initial liquid–liquid sulfur extraction that occurred during the first 10 min for the homogeneous and heterogeneous ECODS catalytic systems, it is possible to observe that this was slightly higher in the absence of solid material, which may have contributed to the lower activity found with the PW_12_@TM–SBA-15 composite. The lower catalytic performance found using the composite may also have been caused by its dispersion between model diesel and the IL phase, unlike the homogeneous IL–PW_12_ that was immobilized in the [BMIM]PF_6_ phase during all ECODS process. 

**Figure 7:**
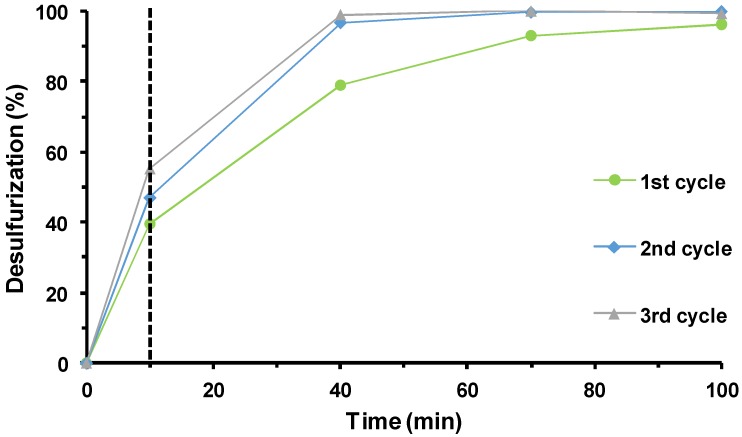
Kinetic desulfurization profiles of multicomponent model diesel catalyzed by PW_12_@TM–SBA-15 for three continuous recycling cycles using ionic liquid ([BMIM]PF_6_) as an extraction solvent at 70 °C.

The continuous reuse of the composite catalyst PW_12_@TM–SBA-15 was performed following the procedure previously described for the reuse of the homogeneous IL–PW_12_. At the end of an ECODS cycle, after stopping the stirring, all solid catalysts remained in the extraction phase and the sulfur-free model diesel was removed and replaced by new sulfurized model diesel and a new aliquot of oxidant. The continuous reusability of the composite was evaluated for three consecutive cycles. The desulfurization profiles for the various cycles are displayed in Figure 7. When comparing the desulfurization performance of the composite catalyst in the three ECODS cycles, some differences were detected, mainly from the first to the consecutive cycles. In particular, an increase in sulfur removal was observed in the second and third cycles when compared with the first cycle. The complete desulfurization of the model diesel was achieved after just 1 h instead of the 1.5 h that was necessary during the first ECODS cycle. This increase observed in the second and consecutive ODS cycles should be related to the presence of previously formed catalytically active peroxo species [39,44,46,59,68].

### 3.3. Catalyst Stability

The stability of the heterogeneous catalyst was evaluated after catalytic use (PW_12_@TM-SBA-15-ac) with different characterization techniques. The vibrational spectra (Figure 1B and Figure S1B in Supplementary Materials) before and after catalysis were similar without significant changes. Both displayed the typical bands assigned to the vibrational modes of PW_12_ and SBA-15 support, suggesting that the main structure of the composite was retained. In the case of FT-Raman, the spectrum after catalytic use displayed some additional bands (marked with an asterisk). These bands are related to the presence of model diesel and related components, as previously observed by our group [44]. These species remain strongly adsorbed to the catalyst, even after the washing procedure, and are most likely the corresponding sulfones of the initial sulfur compounds [69]. The crystalline structure of the SBA-15 support was investigated by powder XRD. The pattern of PW_12_@TM-SBA-15-ac still exhibited the same three main peaks of the hexagonal symmetry of SBA-15 at the same 2θ (Figure 2). Nevertheless, a broadness of the peak indexed to the (100) reflection was observed after catalysis which could be due to a small loss of crystallinity during the consecutive ECODS cycles. The PW_12_@TM-SBA-15-ac material was also studied by SEM/EDS techniques (Figure S3 in Supplementary Materials). The images obtained revealed an identical morphology with the initial composite composed of the same typical elongated structures. Moreover, the EDS analysis revealed the presence of silicon and tungsten at similar relative intensities before and after catalysis. An elemental analysis of the recovered catalyst was also performed to investigate the occurrence of leaching. The results indicated a POM loading of 0.046 mmol/g in the PW_12_@TM–SBA-15-ac composite, corresponding to a leaching of 17%. Such a low value is most likely related to the interactions established between the amine groups and PW_12_ that help to keep POM molecules in the composite during catalytic use. The characterization of PW_12_@TM–SBA-15-ac shows that the heterogeneous catalyst was stable under the experimental ECODS conditions and retained its main structure and chemical composition.

The integrity of the homogeneous [BPy]_3_PW_12_ catalyst was assessed by ^31^P NMR. The spectra of the starting catalyst after one and three ECODS cycles are represented in Figure S4 in the Supplementary Materials. The spectrum of [BPy]_3_PW_12_ before catalytic use exhibited a single peak at *δ* = −13.89 ppm. The recovered catalyst after the first ECODS cycle using the biphasic system model diesel/MeCN also presented a ^31^P NMR spectrum with the initial single peak at −13.89 ppm. This result indicates that no formation of active peroxo-species occurred, which can explain the absence of catalytic activity observed in this homogeneous system. Regarding the ^31^P NMR spectra obtained after the first and the third ECODS cycles using the model diesel/[BMIMI]PF_6_ system, these exhibited three main peaks located at *δ* = 1.86, −2.91 and −8.52 ppm. These species should correspond to peroxo-complexes (known as PW_x_O_y_) with different P/W ratios which are formed during the decomposition of the Keggin structure in the presence of H_2_O_2_ [28,37,54,70,71]. The interaction between POM and the IL [BMIMI]PF_6_ can also promote shifts in the ^31^P signals of the known peroxo-complexes which makes it extremely difficult to perform unequivocal assignment. For instance, Liu et al. reported alkene epoxidation using a PW_12_-based catalyst and ionic liquids as solvents. After catalytic use, an additional ^31^P signal at *δ* = −8.6 ppm was observed which the authors were unable to identify [70]. Interestingly, the intensity of the peak at a higher chemical shift (1.86 ppm) increased along the ECODS cycles when compared with the intensities of the other two peaks. At the end of the third cycle, the peak at *δ* = 1.86 became the main peak in the ^31^P spectrum and should therefore correspond to the most active species in the studied reaction.

## 4. Conclusions

In this work, various ionic–liquid Keggin-type phosphotungstate compounds were prepared using 1-butyl-3-methylimidazolium cation [BMIM]_3_PW_12_, 1-butylpyridinium cation [BPy]_3_PW_12_ and hexadecylpyridinium cation [HDPy]_3_PW_12_. These compounds showed high catalytic activity during the desulfurization of a multicomponent model diesel (total desulfurization after 1 h). This diesel was treated efficiently in two main steps: initial liquid–liquid sulfur extraction and catalytic sulfur oxidation (ECODS) using a low excess of H_2_O_2_ oxidant (H_2_O_2_/S = 8) at 70 °C. The catalytic performance of these homogeneous catalysts was higher in the present of the biphasic system 1:1 diesel/ionic liquid [BMIM]PF_6_ than in the 1:1 diesel/acetonitrile system. In the first ECODS system, the high activity of the Keggin catalysts was due to their decomposition into different peroxo-compounds. The various ionic liquids cations used in the homogeneous catalysts did not confer different catalytic performances. Furthermore, the continuous recycling of the extraction [BMIM]PF_6_ phase containing the homogeneous catalyst caused some loss of oxidative catalytic activity after the first ECODS cycle. The disadvantages associated with the homogeneous catalytic systems were overcome by the application of the PW_12_@TM–SBA-15 heterogeneous catalyst, prepared by the immobilization of the same PW_12_ catalytic center on the trimethylammonium functionalized-SBA-15. In this case, the solid catalyst presented a similar oxidative desulfurization efficiency using acetonitrile or ionic liquid [BMIM]PF_6_ solvents. On the other hand, similar catalytic performances of the composite and IL-PW_12_ homogeneous compounds were found, resulting in complete desulfurization after approximately 1 h. Moreover, the high recycle capacity of the composite was observed, whereby the ionic liquid solvent and the solid catalysts were reused together for consecutive ECODS cycles, and an increase in oxidative desulfurization efficiency was observed after the first cycle. At the end, the solid catalytic composite was isolated, and its structural stability was confirmed. Therefore, the high catalytic performance obtained with the PW_12_@TM–SBA-15 composite indicates that the trimethylammonium–SBA-15 support confers an optimal environment for promoting efficient catalytic sulfur oxidation, ensuring its activity and robustness. 

## Supplementary Materials

The following are available online at http://www.mdpi.com/1996-1944/11/7/1196/s1, Figure S1: FT-IR spectra of (A) the PW_12_-hybrids and (B) the starting SBA-15 support, the functionalized TMA-SBA-15 and the corresponding PW_12_@TM-SBA-15 composite before and after catalysis. Figure S2: Kinetic profile for the blank experiment (without catalyst) using the ECODS system model diesel/[BMIM]PF_6_. Figure S3: SEM images of the PW_12_@TM-SBA-15-ac material at different magnifications: (A) ×5000, (B) ×25000, (C) ×60000 and (D) EDS spectrum. Figure S4: ^31^P NMR spectra of [BPy]_3_PW_12_ before and after catalytic use (ac) using MeCN or IL. Figure S5: ^31^P NMR spectrum of ionic liquid phase after catalytic use upon PW_12_@TM-SBA-15 catalyst removal. Table S1: Individual and total desulfurization percentages after the initial extraction from the multicomponent model diesel to the extractant phase (MeCN or IL) using TM-SBA-15, [BPy]_3_PW_12_ and PW_12_@TM-SBA-15 as catalysts.
