# Supplementary material for: Improving the Catalytic Performance of Keggin [PW12O40]3− for Oxidative Desulfurization: Ionic Liquids versus SBA-15 Composite
Source: Materials (Basel). 2018 Jul 12;11(7):1196. doi: 10.3390/ma11071196 (PMC6073681; doi:10.3390/ma11071196)
Supplement: Supplementary file 1 [file materials-11-01196-s001.pdf]

Supplementary Materials: Improving the Catalytic Performance of Keggin [PW12O40]3− for Oxidative Desulfurization: Ionic Liquids versus SBA-15 Composite

Susana O. Ribeiro, Beatriz Duarte, Baltazar de Castro, Carlos M. Granadeiro and Salete S. Balula

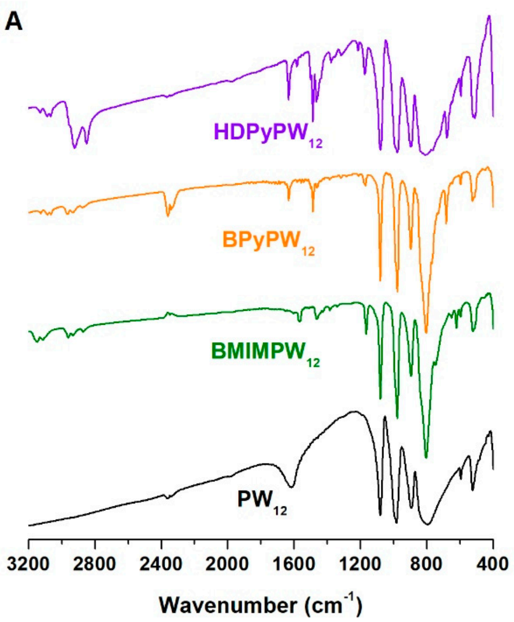

Figure S1. FT-IR spectra of (A) the PW_{12}-hybrids and (B) the starting SBA-15 support, the functionalized TMA-SBA-15 and the corresponding PW_{12}@TM-SBA-15 composite before and after catalysis.

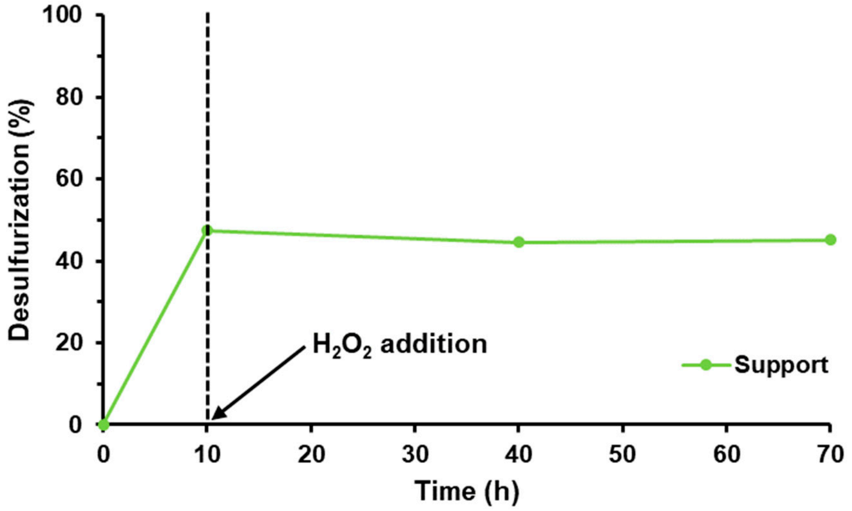

Figure S2. Kinetic profile for the blank experiment (without catalyst) using the ECODS system model diesel/[BMIM]PF_6.

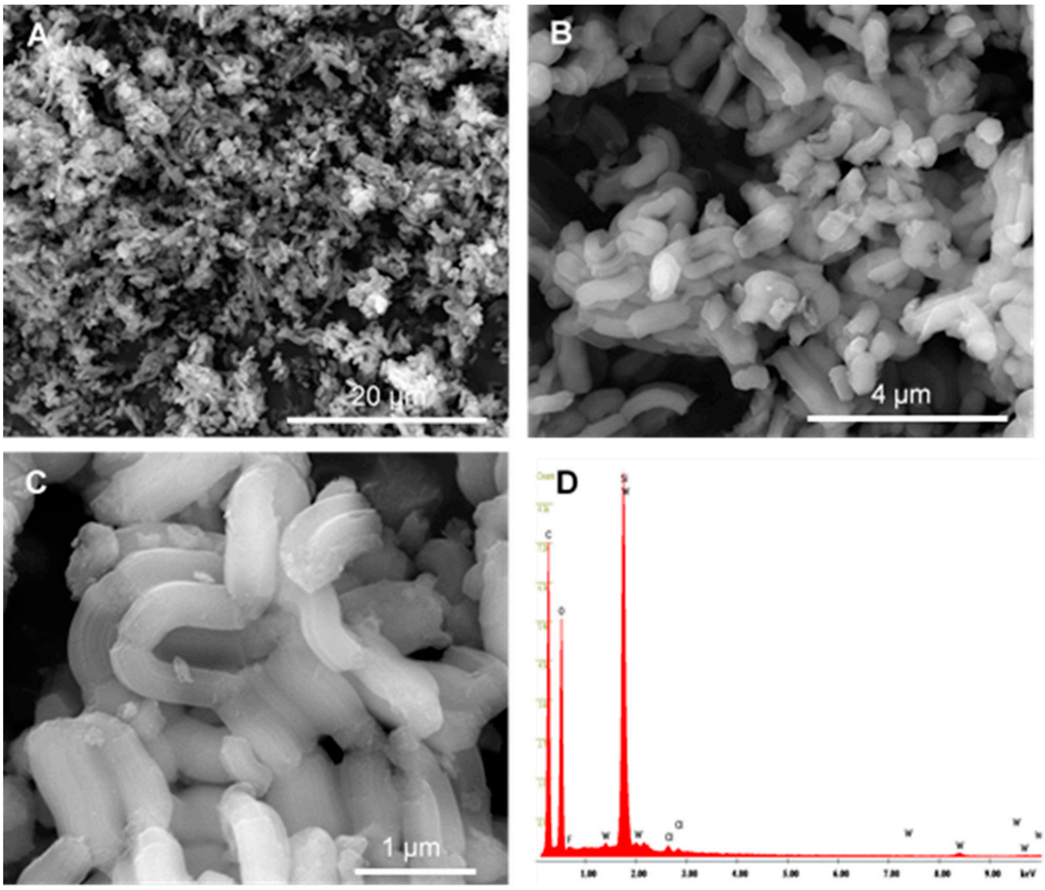

Figure S3. SEM images of the PW_{12}@TM-SBA-15-ac material at different magnifications: (A) ×5000, (B) ×25000, (C) ×60000 and (D) EDS spectrum.

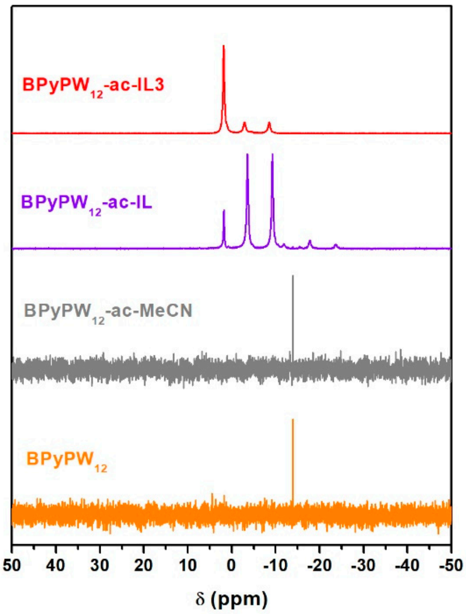

Figure S4. ^{31}P NMR spectra of $[BPy]_3PW_{12}$ before and after catalytic use (ac) using MeCN or IL.

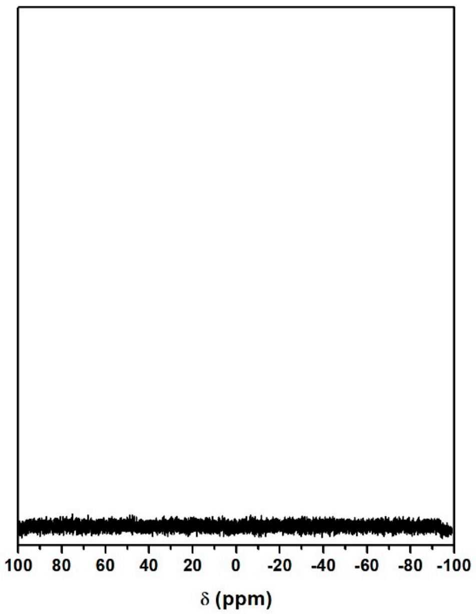

Figure S5. ^{31}P NMR spectrum of ionic liquid phase after catalytic use upon PW_{12}@TM-SBA-15 catalyst removal.

Table S1. Individual and total desulfurization percentages after the initial extraction from the multicomponent model diesel to the extractant phase (MeCN or IL) using TM-SBA-15, $[BPy]_3PW_{12}$ and PW_{12}@TM-SBA-15 as catalysts.

		Desulfurization (%)				
**Catalyst**	**Solvent**	**1-BT**	**DBT**	**4-MDBT**	**4,6-DMDBT**	**TOTAL**
TM-SBA-15	MeCN	63	66	61	56	62
	IL	53	55	45	37	47
$[BPy]_3PW_{12}$	MeCN	61	65	60	54	60
	IL	66	58	45	34	51
PW_{12}@TM-SBA-15	MeCN	63	62	55	47	57
	IL	54	50	34	21	40
